# Identification and Characterization of a Novel Thermostable and Salt-Tolerant β-1,3 Xylanase from *Flammeovirga pacifica* Strain WPAGA1

**DOI:** 10.3390/biom10091287

**Published:** 2020-09-07

**Authors:** Zhiwei Yi, Zhengwen Cai, Bo Zeng, Runying Zeng, Guangya Zhang

**Affiliations:** 1 Department of Bioengineering and Biotechnology, Huaqiao University, Xiamen 361021, China; yizhiwei@tio.org.cn (Z.Y.); 1511315006@stu.hqu.edu.cn (Z.C.); 19013087005@stu.hqu.edu.cn (B.Z.); 2 Technology Innovation Center for Exploitation of Marine Biological Resources, Third Institute of Oceanography, Ministry of Natural Resources, Xiamen 361005, China; zeng@tio.org.cn

**Keywords:** *Flammeovirga pacifica*, β-1,3 Xylanase, thermophily and salt tolerance, molecular dynamics

## Abstract

β-1,3 xylanase is an important enzyme in the biorefinery process for some algae. The discovery and characterization of new β-1,3 xylanase is a hot research topic. In this paper, a novel β-1,3 xylanase (Xyl88) is revealed from the annotated genome of *Flammeovirga pacifica* strain WPAGA1. Bioinformatic analysis shows that Xyl88 belongs to the glycoside hydrolase 26 (GH26) with a suspected CBM (carbohydrate-binding module) sequence. The activity of rXyl88 is 75% of the highest enzyme activity (1.5 mol/L NaCl) in 3 mol/L NaCl buffer, which suggests good salt tolerance of rXy188. The optimum reaction temperature in the buffer without NaCl and with 1.5 mol/L NaCl is 45 °C and 55 °C, respectively. Notably, the catalytic efficiency of rXyl88 (*k_cat_/K_m_*) is approximately 20 higher than that of the thermophilic β-1,3 xylanase that has the highest catalytic efficiency. Xyl88 in this study becomes the most efficient enzyme ever found, and it is also the first reported moderately thermophilic and salt-tolerant β-1,3 xylanase. Results of molecular dynamics simulation further prove the excellent thermal stability of Xyl88. Moreover, according to the predicted 3D structure of the Xyl88, the surface of the enzyme is distributed with more negative charges, which is related to its salt tolerance, and significantly more hydrogen bonds and Van der Waals force between the intramolecular residues, which is related to its thermal stability.

## 1. Introduction

As the widely distributed primary producer in the ocean, seaweed produces about half of the world’s primary production. Seaweed biorefinery has become a research hotspot for scholars all over the world [1]. Cell walls of most algae are utterly free of cellulose. However, they rely on the polysaccharide structure to maintain the mechanical strength of the cell wall. The most widely distributed component of brown algae is alginate, a cell wall polysaccharide composed of polymeric blocks of α-(1,4) *O*-linked β-d-mannuronate (M)^2^ and its C5 epimer α-l-guluronate (G) [2]. Agar is a phycocolloid extracted from the cell wall of a group of red algae (Rhodophyceae) including Gelidium and Gracilaria, which is composed of repetitive units of β-d-galactose and 3,6-anhydro-α-l-galactose [3]. Carrageenan is derived from a number of seaweeds of the class Rhodophyceae. It is a sulfated polygalactan with 15 to 40% of ester-sulfate content and an average relative molecular mass well above 100 kDa, and is formed by alternate units of d-galactose and 3,6-anhydro-galactose (3,6-AG) joined by α-1,3 and β-1,4-glycosidic linkage [4]. Cellulose is a highly homogeneous polymer that consists of β-1,4-linked glucose units, which make interstrand hydrogen bonds to form a highly stable crystalline lattice [5]. Chitin is a natural polysaccharide of major importance, which is formed by poly (β-(1→4)-N-acetyl-d-glucosamine) [6,7]. β-1,4-Xylan consists of a linear polymer of β-(1,4)-linked xylose residues substituted with acetyl, glucuronic acid (GlcA), 4-*O*-methylglucuronic acid (Me-GlcA), and arabinose residues [8]. β-1,3-Xylan, a homopolysaccharide of β-1,3-linked d-xylopyranose units [9], is derived from the cell walls of red alga (Porphyra) [10] and green alga (Caulerpa) [11].

Xylanase can decompose hemicellulose, arabinoxylan and plant fiber, which means xylanase can be used in bread making, animal feed and the textile industry [12,13]. Enzymatic hydrolysis of β-1,3 xylan can efficiently obtain different oligomeric polysaccharides and sugars, such as β-Dxylopyranosyl units. Polysaccharides can induce apoptosis of cancer cells [14], whereas β-Dxylopyranosyl can be converted into ethanol [15,16] and valuable 2, 3-butanediol, furfural, xylitol, and other essential chemicals. There are 5 to 20% of β-d-xylopyranosyl residues among the sugars used in the production of ethyl alcohol [13]. Xylitol is a polyalcohol with a sweetening power comparable to that of sucrose [17]. It is a non-cariogenic sweetener, suitable for diabetic and obese individuals and is recommended for the prevention of osteoporosis and respiratory infections, lipid metabolism disorders, and kidney and parenteral lesions. Various commercial products containing xylitol, such as chewing gum, are fashionable on the market [18]. Therefore, using β-1,3 xylanase to treat seaweed properly can obtain raw materials for new energy production and functional foods with health effects, which can consequently improve the economic value of seaweed.

The study of β-1,3 xylanase (EC 3.2.1.32) dates back to the 1960s, when Iriki, Y. and his team first discovered that several fungi could secrete enzymes that hydrolyzed β-1,3 xylan [19]. Similar to cellulose and hemicellulose, β-1,3 xylanase consists of a catalytic domain (GH26), a glycine-rich linkage structure, with or without carbohydrate-binding modules (CBM). CBM is the family 31 (CBM31), and CBM6 also exists [20]. These CBMs have a specific affinity for insoluble β-1,3 xylan and can effectively bind non-aqueous β-1,3 xylan to improve catalytic efficiency [21,22]. Studies have shown that hydrolysis and catalysis likely follow the ^1^S_3_ → ^4^H_3_ → ^4^C_1_ conformational pathway [9]. Compared with the well-known β-1,4 xylanase (EC 3.2.1.8), research on β-1,3 xylanase is at the preliminary stage, and only five β-1,3 xylanase genes are expressed in *Escherichia coli* and characterized to date (Table S1).

Based on the annotated *Flammeovirga pacifica* strain genome (Accession: PRJNA263711), a suspected β-1,3 xylanase gene (Xyl88) was collected and cloned into *Escherichia coli* BL21 (DE3) for expression. After purification, characterization was processed, and optimal reaction temperatures were measured as 45 °C–55 °C, which can tolerate NaCl concentrations up to 5 mol/L. The moderately thermophilic and salt-tolerant properties were found for the first time in β-1,3 xylanase, whereas rXyl88 performed the highest catalytic efficiency among the currently reported β-1,3 xylanases. Xyl88 has the potential to fully exploit the value of seaweed in industrial applications, such as the energy industry and food industry.

## 2. Materials and Methods

### 2.1. Preparation of β-1,3 Xylan and β-1,3 Oligosaccharides

β-1,3 xylan was prepared from a green algae (Caulerpa racemosa var. laetevirens) with reference to the method of Iriki et al., [19] and the hydroxy alcohol β-1,3 xylan was obtained through the reaction of the above-mentioned insoluble β-1,3 xylan and ichloroethanol under certain conditions according to the research by Yamaura et al. [23]. Total sugar was determined by the phenol sulfuric acid method [24], and the content was above 95%, which could be used as the substrate for the subsequent study on enzymatic properties. Then, β-1,3 xylan was randomly hydrolyzed by trifluoroacetic acid at 70 °C for 3 h to obtain β-1,3 xylooligosaccharides for subsequent control study on products using thin-layer chromatography [25].

### 2.2. Plasmid Construction

The Xyl88 protein sequence was translated via the nucleotide sequence of the *Flammeovirga pacifica* strain genome (locus = Scaffold1:3919036:3920313:+), which was numbered A0A1S1Z2X9 in the Uniprot database. Then, six histidines were designed to its N-terminus as a tag for subsequent purification, and the gene with optimized codons was synthesized at Genewiz (Suzhou, China). After double digestion with NedI and HindIII, the plasmid was ligated into the expression vector pET 22b (Takara, Dalian, China). Finally, the plasmid containing Xyl88 gene was introduced into *Escherichia coli* BL21 (DE3; SANGON, Shanghai, China) for expression.

### 2.3. SDS-Polyacrylamide Gel Electrophoresis (SDS-PAGE) with Precast Gels

A total of 10 μL rXyl88 was added into tube and mixed with 2.5 μL 5-fold concentrated loading buffer (235 mM SDS, 33.6% glycerol *v*/*v*, 5% 2-mercaptoethanol *v*/*v*, 0.67% bromophenol blue *w*/*v*, 210 mM Tris–HCl, pH 6.8). After 5 min at 95 °C in a water bath, mixed rXyl88 was prepared. Then, 5 μL Marker (TheromFisher scientific, catalog number: 26616) and 10 μL prepared rXyl88 were noded into NuPAGE Bis-Tris acrylamide 12% precast gels (Thermo Fisher Scientific, Beijing, China), respectively. The samples were electrophoresed at 80 V until the dye front reached the top of the separating gel (20 min). The voltage was increased to 120 V and continued to run until the bottom of the gel was reached (about 1 h).

### 2.4. Expression and Purification of Xyl88 Recombinant Protein

The *Escherichia coli* strain containing the Xyl88 gene was cultured in an LB medium with an ampicillin sodium concentration of 100 μg/m at 37 °C (200× *g*) for 12 h and then inoculated into TB medium at 37 °C. At an OD_600_ of 0.5–0.6, the IPTG at a final concentration of 0.2 mmol/L was added to the medium to induce the expression at 18 °C and 160× *g* for 24 h. *Escherichia coli* was collected by centrifugation at 4 °C and 8000× *g* for 15 min in a cryogenic centrifuge. Then, the thallus was resuspended in a 20 mmol/L phosphate-buffered saline (Na_2_HPO_4_•12H_2_O/NaH_2_PO_4_•2H_2_O, pH 7.5) and ultra-sonicated in an ice bath. The cell debris was removed by centrifugation at 4 °C and 12,000× *g* for 10 min. Finally, the target protein was separated and purified using a nickel affinity chromatography column (1.4 cm × 6.5 cm, Smart-Lifesciences, Changzhou, China).

The molecular weight and purity of the obtained rXyl88 recombinant protein were identified using denaturing gel electrophoresis, and the protein concentration was measured by the Coomassie Brilliant Blue method [26]. Subsequently, the purified protein was hydrolyzed by trypsin, and the library sequence was identified and analyzed using LC-ESI-QUAD-TOF to compare whether the target protein sequence was consistent with the theoretical sequence.

### 2.5. Enzymatic Activity Measurement

The rXyl88 activity was measured by the DNS method with xylose as the standard [27]. The whole reaction system contained 350 μL of 10 g/L hydroxy alcohol β-1,3 xylan dissolved in 20 mmol/L pH 7.5 phosphate buffer and 50 μL of purified enzyme protein solution at a concentration of 0.213 g/L. Then, the system was in a water bath at 45 °C for 5 min, and then immediately added with 400 μL DNS reagent to terminate the reaction and turned to a boiling water bath for 5 min. After cooling, 1.6 mL of deionized water was added to measure light absorption at a wavelength of 540 nm. The amount of enzyme required to produce 1 μmol of reducing sugar per minute was defined as one unit of enzyme activity, and all enzymatic properties were measured in triplicate.

### 2.6. Enzymatic Property Measurement

Considering that Xyl88 is a marine-derived enzyme, its salt tolerance was first evaluated. Under the above-mentioned conditions for enzymatic activity measurement, NaCl (0–5 mol/L) at different concentrations was set, followed by the measurement of its optimum reaction temperature with and without 1.5 mol/L NaCl (20 °C–75 °C). Moreover, the optimal pH was determined by changing the buffer and pH (20 mmol/L Na_2_HPO_4_/citric acid: pH 3.0−8.0 and 20 mmol/L Tris/HCl: pH 7.5−10.0) under the same conditions.

rXyl88 was in a water bath at 40 °C, 45 °C, and 50 °C to measure the thermal stability of rXyl88 with and without 1.5 mol/L NaCl, and the residual enzyme activity was measured every 15 min under optimum conditions for 90 min. An enzymatic solution was first incubated at 45 °C for 15 min to understand the effect of NaCl on the thermal stability of Xyl88, and then its residual enzyme activity was measured. The solution was quickly split into two parts, of which one was added to NaCl at a final concentration of 1.5 mol/L, and the other remained unchanged. The water bath continued with the experimental condition similar to that of the thermal stability measurement.

Tris/HCl buffer solution was used to dissolve the substrate and rXyl88 under the optimal reaction conditions, and Na^+^, K^+^, Zn^2+^, Mn^2+^, Ba^2+^, Mg^2+^, Ni^2+^, Cu^2+^, Ca^2+^, Co^2+^, Fe^2+^, Fe^3+^, and EDTA were added with a final concentration of 10 mmol/L to determine the effect of metal ions and EDTA on the enzymatic activity of rXyl88.

Finally, insoluble β-1,3 xylan and soluble hydroxy alcohol β-1,3 xylan solutions were prepared with and without 1.5 mol/L NaCl, and the concentration was adjusted to 1.0–10.0 mg/mL. The kinetic constant of Xyl88 was measured under optimal reaction conditions, and the *K_m_* and maximum reaction rate *V_max_* were calculated by fitting the Michaelis–Menten equation curve.

All experiments were set up three times.

### 2.7. TLC analysis of rXyl88 Hydrolysate

The hydrolysate was analyzed by thin-layer chromatography. First, 350 μL of 1% hydroxy alcohol β-1,3 xylan was added to 50 μL of appropriately diluted enzyme solution and removed after different reaction times under optimum reaction conditions. The enzyme was inactivated to terminate the reaction in the boiling water bath for 10 min and then centrifuged at 12,000× *g* for 10 min at room temperature. The supernatant enzymatic hydrolysate was spotted onto a thin-layer chromatography plate (silica gel 60, Merck, Darmstadt, Germany). The developing solvent (proportion of n-butanol:acetic acid:water volume [10:5:1]) was used for development analysis. After completion, the color developing agent [28] (diphenylamine/aniline/phosphoric acid) was uniformly sprayed, and the plate was placed in an oven at 100 °C for 10 min to develop color.

### 2.8. Protein Sequence-Structure Analysis

First, the conserved sequence of the protein was analyzed using the NCBI CONSERVED Domain Architecture Retrieval Tool [29]. A total of 50 protein sequences annotated as β-1,3 xylanase were downloaded from UniProt. Clustal Omega 1.2.2 (http://www.clustal.org/) was used for multiple sequence alignment. SWISS-MODEL [30], I-TASSER [31], and Robetta [32], three server bases, were used to construct the 3D structure of the enzyme, and MolProbity [33] was used for the structural assessment of the model (Tables S2–S4). The model derived from the beginning on the Robetta server was the most reliable because the enzyme sequence failed to find a structure with an identity higher than 30% from the PDB database. Finally, the analysis was processed using Pymol and UCSF Chimera [34,35].

Then, the RING server [36] was used to calculate and analyze the interaction network between the room-temperature β-1,3 xylanase rXYL4 (PDB ID: 2ddx [37]) and Xyl88 protein structural residues, which were from the *Vibrio* sp. strain AX-4. Hydrogen bond, Van der Waals force, disulfide bond, salt bridge, π–π stacking force, and π cation cutoff distance were set to 3.5 Å, 0.5 Å, 2.5 Å, 4.0 Å, 6.5 Å, and 5.0 Å, respectively, and NAMD (Git-2017-11-06 for Linux-x86_64-multicore-CUDA [38]) and VMD [39] were used to calculate the number of hydrogen bonds between protein and water solution at different temperatures after energy minimization.

Finally, the molecular dynamics simulation was carried out by the CHARMM force field via NAMD in a constant-temperature and constant-pressure system (NPT) [40]. The step size was 2 fs, and the energy was optimized to a minimum of 2000 steps, with 5 ns as a simulation time unit until the protein was depolymerized. Root-mean-square deviation (RMSD) was calculated by VMD, and the molecular simulation trajectory was observed. The thermal tolerance of rXYL4 and rXyl88 at 530 K and 540 K, respectively, was analyzed.

## 3. Results

### 3.1. Expression and Purification of Recombinant Protein rXyl88

**Figure 1:**
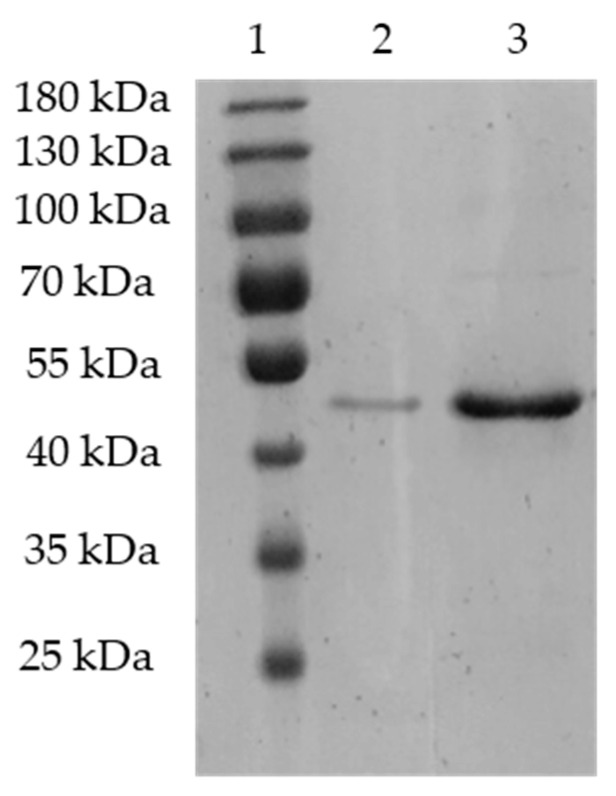
The SDS-PAGE of purified recombination Xyl88. Lane 1: protein molecular weight marker; lane 2: the purified recombination rXyl88 with 10-fold dilution, and lane 3: the purified recombination rXyl88.

**Figure 2:**
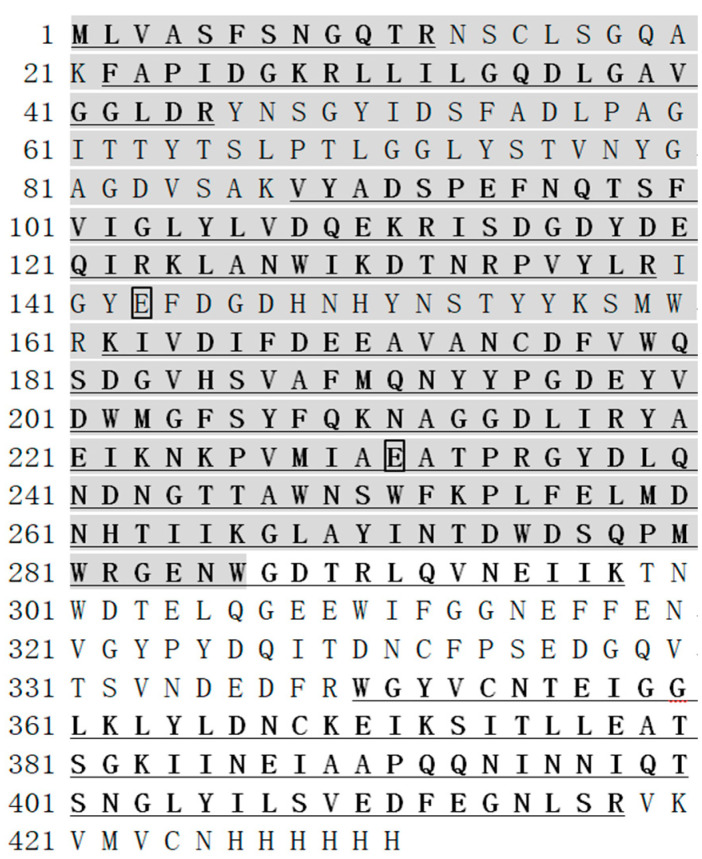
The sequence of the Xyl88. The LC-ESI-QUAD-TOF analysis results are underlined and in bold, the conserved GH26 catalytic domain shaded in gray and the catalytic residues are marked with a box.

The *Escherichia coli* fused with Xyl88 was fermented and cultured, then induced by IPTG, and purified by nickel column. The crude enzyme solution and the obtained target protein eluant were subjected to SDS-PAGE verification. The results are shown in Figure 1, which shows a distinct band in 40–55 kDa. The result was consistent with the theoretically calculated molecular weight of 48.2 kDa via ProtParam (http://web.expasy.org/protparam/), and the bands purified by nickel column in lanes 2 and 3 were relatively simple. The Xyl88 recombinant protein band in SDS-PAGE was subjected to gel extraction and then identified by mass spectrometry after trypsin digestion. The molecular weight was measured as 49,296 Da by UHPLC–QTOF (Agilent 1290/6545), similar to the electrophoresis outcome and theoretically calculated molecular weight. On the basis of the Xyl88 amino acid sequence, the search library was constructed. The peptide sequencing showed that the coverage was 68%, shown as bold and underlined amino acids in Figure 2. The area verified by the sequencing covered the gray-shaded part of the GH26 functional domain sequence, particularly the two active sites (marked block). This result indicated that the expressed protein was the expected rXyl88 protein, which has high purity and could be used for subsequent experiments and studies.

### 3.2. Measurement of rXyl88 Enzymatic Properties

**Figure 3:**
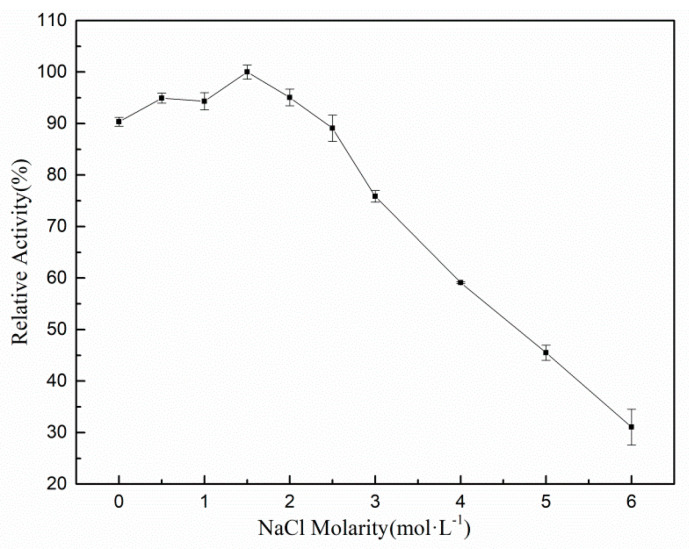
The influences of NaCl on the activity of the Xyl88.

Since rXyl88 was derived from marine bacteria, its salt tolerance was analyzed. Figure 3 showed that as the NaCl concentration increased, the enzymatic activity also showed a gently upward trend, and began to decrease when the NaCl concentration reached 1.5 mol/L, indicating that the optimum NaCl concentration was 1.5 mol/L. In addition, the activity of rXyl88 in NaCl-free buffer was close to the optimum NaCl concentration, which was about 90%. When the salt concentration reached 3 mol/L, rXyl88 could still maintain about 80% of the highest activity. The above-mentioned results indicated that rXyl88 was less dependent on NaCl but could also tolerate relatively high concentrations of NaCl.

**Figure 4:**
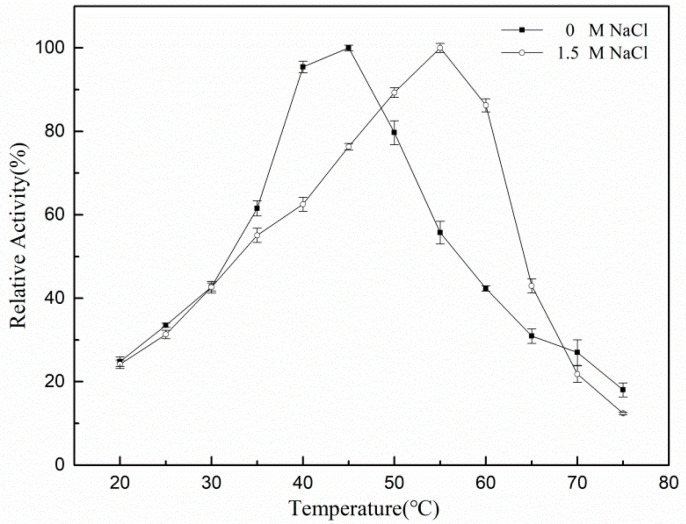
The influences of temperature on the activity of the Xyl88.

The substrates with 0 and 1.5 mol/L NaCl concentration were prepared separately to determine whether the optimum reaction temperature of the enzyme was affected by the concentration of NaCl at pH 7.5 and 20 °C–75 °C, and the enzyme was reacted in the substrates for 5 min. The optimum reaction temperature of rXyl88 measured is shown in Figure 4.

In the absence of NaCl, the enzyme activity increased with temperature, reaching its maximum at 45 °C. Then, the enzymatic activity began to decrease, indicating that 45 °C was the optimum reaction temperature for rXyl88. With 1.5 mol/L NaCl, the optimum reaction temperature increased to 55 °C, which was 10 °C higher than the former.

**Figure 5:**
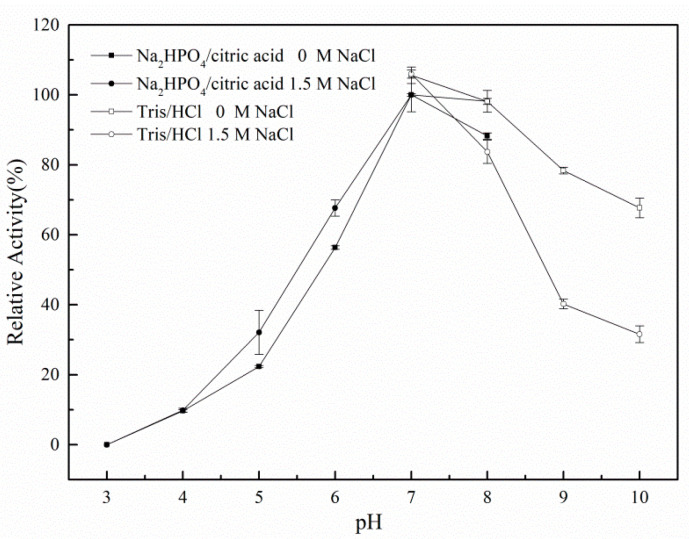
The influences of pH on the activity of the Xyl88.

A 1% hydroxy alcohol β-1,3 xylan solution was prepared using buffers of different pH. The solution was divided into two groups: the first group had no NaCl, whereas the other group contained NaCl at a concentration of 1.5 mol/L. As shown in Figure 5, rXyl88 has basically the same activity in catalyzing the substrates under the two conditions. The enzyme activity was low under acidic conditions, and the enzymatic activity increased with the gradual increase of pH until reaching the neutral status. The optimum reaction pH was between 7.0 and 8.0, and then a certain degree of decline of activity was observed. NaCl had little effect on the optimum reaction pH of the enzyme.

**Figure 6:**
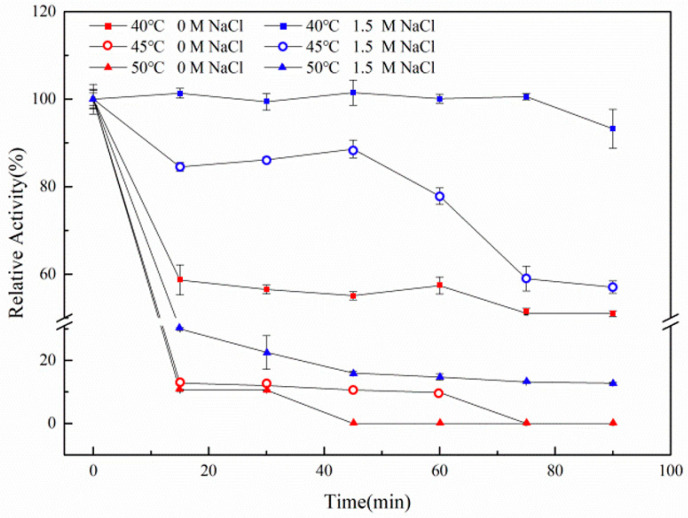
The influences of temperature on the stability of rXyl88.

The thermal stability of the 1,3-xylanase in a buffer containing no NaCl and containing 1.5 mol/L NaCl was measured. The results shown in Figure 6 indicated that rXyl88 was sensitive to temperature in a NaCl-free reaction system. After incubation for 15 min at 40 °C, the activity loss was massive, and only about 60% of the activity remained. Then, the activity of Xyl88 showed no significant change with the extension of incubation time. At 45 °C and 50 °C, the rXyl88 enzyme activity was gradually lost until it was denatured and inactivated, indicating that rXyl88 was less thermally stable in the absence of NaCl. However, the thermal stability of Xyl88 was significantly improved in a 1.5 mol/L NaCl reaction system. No significant loss of enzymatic activity at 40 °C was detected, and although the temperature was increased to 45 °C and 50 °C, the enzymatic activity loss was not as severe as that without NaCl. For example, after incubation at 45 °C for 60 min, the enzyme still maintained about 80% of the activity in the reaction solution containing 1.5 mol/L NaCl, whereas 1,3-xylanase in the reaction solution containing no NaCl retained only about 10% of the activity. Therefore, NaCl has a significant influence on the thermal stability of the enzyme, and rXyl88 has a specific dependence on NaCl.

**Figure 7:**
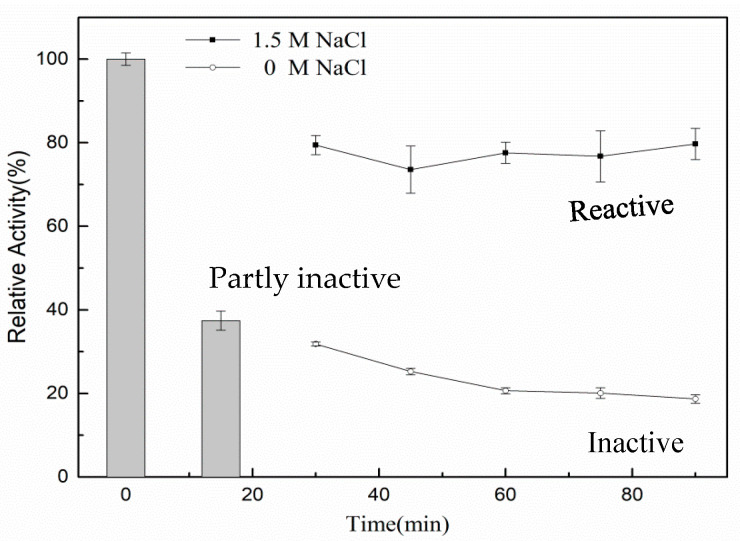
The influences of NaCl on the thermostability of the rXyl88. The activity of rXy188 was detected at 45 °C. Bars show the relative activity of rXyl88 in the initial time and the 15th min after incubation, respectively. The lines with disc symbol and hollow circle were carried out without supplementary of NaCl and in the presence of 1.5 mol/L NaCl, respectively. All assays were performed in triplicate, and standard deviations are shown with error bars.

The enzyme was incubated for 15 min in a 45 °C water bath to understand the effect of NaCl on the thermal stability of rXyl88. The reaction solution was then divided into two parts: one part with NaCl solution at a final concentration of 1.5 mol/L and the other part without NaCl. Then, incubation continued at the same temperature, and the change of enzymatic activity was measured. The results in Figure 7 indicated that the activity of NaCl-free enzyme solution gradually decreased with incubation time until the activity was about 20% of the initial state. By contrast, the enzymatic activity immediately recovered from about 40% to 80% after incubation in the 1.5 mol/L NaCl reaction solution for 15 min and remained basically unchanged. NaCl could partially renature the 1,3-xylanase and reactivate it at high catalytic activity.

The effects of 10 mmol/L metal ions and EDTA on the activity of rXyl88 (Table 1) indicated that Zn^2+^, Mn^2+^, Ni^2+^, Cu^2+^, Co^2+^, and Fe^3+^ have strong inhibitory effects on the enzymatic activities. The relative enzymatic activities were all below 50%. Cu^2+^ (18.73%) and Fe^3+^ (13.72%) have the strongest inhibition, whereas Na^+^ (116.35%) and Mg^2+^ (117.63%) could properly improve the catalytic activity of rXyl88. Therefore, this enzyme was sensitive to metal ions, similar to the β-1,3 xylanase derived from *Vibrio* sp. XY-214 [41]. However, β-1,3 xylanases have some differences. For example, Ba^2+^, Ca^2+^, Mg^2+^, Na^+^, and Zn^2+^ have little effect on the activity of β-1,3 xylanase derived from *Alcaligenes* sp. XY-234 [42], whereas Ca^2+^, Mg^2+^, and EDTA have a minor effect on the activity of β-1,3 xylanase derived from *Vibrio* sp. strain AX-4 [43] and *Thermotoga neapolitana* strain DSM 4359 [44].

The catalytic kinetic constant of rXyl88 measured under the optimum reaction conditions (Table 2) indicated that the presence or absence of NaCl has little effect on the affinity of the enzyme and the substrate and has no noticeable impact on the catalytic efficiency of the enzyme. However, this enzyme has a stronger affinity for the soluble substrate as a whole. With regard to $k_{cat}/K_{m}$, given soluble glycol β-1,3-xylan as the substrate for the enzyme, the catalytic ability was four times of that of the insoluble substrate.

**Figure 8:**
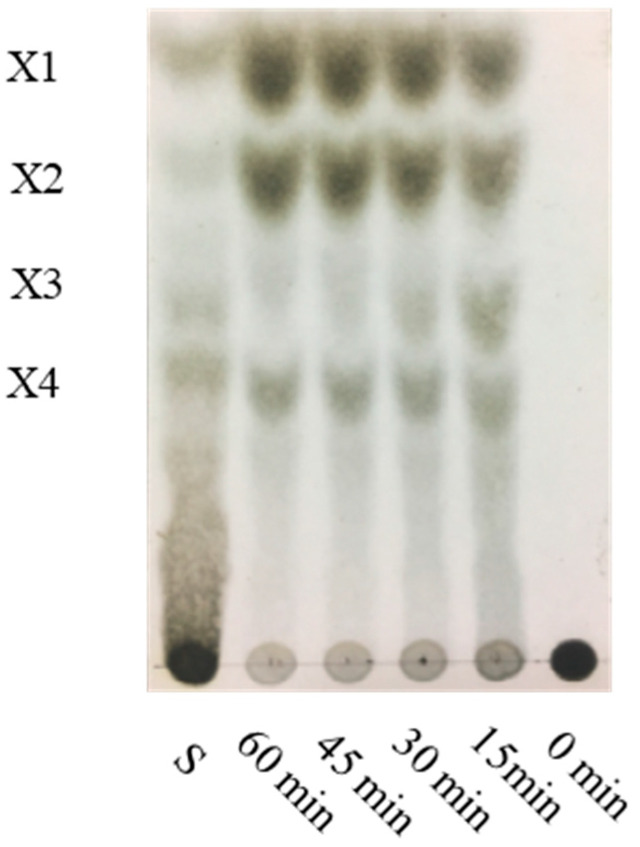
Hydrolysis of β-1,3-xylan by the rXyl88 with different hydrolysis time. Lane S, markers: X1 xylose, X2 β-1,3-xylobiose, X3 β-1,3-xylotriose, X4 β-1,3-xylotetraose, obtained by hydrolysis of β-1,3-xylan by TFA.

Thin-layer chromatography was used to analyze the composition of the products obtained after the hydrolysis of rXyl88 with different hydrolysis time. As the hydrolysis time extends, the product content also increases (Figure 8). A noticeable increase in the yield of the products from xylose to xylotetraose was found after 15 min hydrolysis. The content of xylotetraose was still increasing, whereas the content of xylotriose was lower. The products were xylose and xylobiose. Given the high catalytic efficiency of rXyl88, xylan was hydrolyzed into the product, which almost composed of monosaccharides and disaccharides in 1 h.

### 3.3. Thermophilic Salt-Tolerant Mechanism of β-1,3 Xylanase

The above-mentioned experiments showed that the obtained β-1,3 xylanase (rXyl88) was a moderately thermophilic salt-tolerant enzyme, which was the first reported enzyme with this characteristic in this hydrolase family. Through bioinformatics, the characteristics of its sequence and structure were analyzed, which provided a reference for understanding its extremophile mechanism.

**Figure 9:**
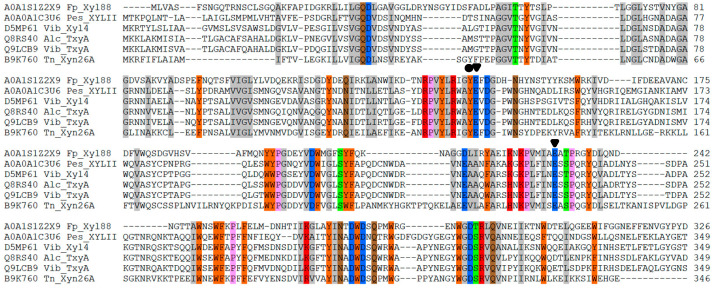
Multiple sequences alignment of biochemically characterized β-1,3-xylanases catalytic domain with Xyl88.

**Figure 10:**
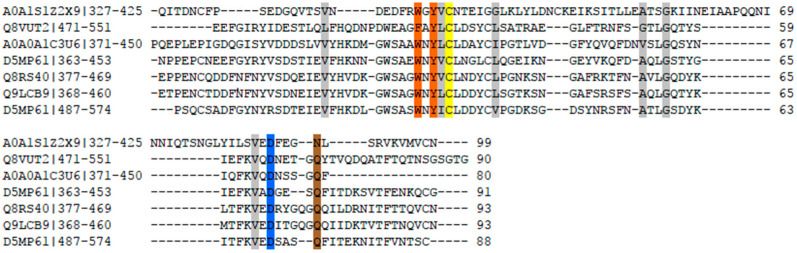
Multiple sequence alignment of CBM (carbohydrate-binding module) with Xyl88 hypothesis CBM.

First, its sequence analysis showed that the amino acid sequences of β-1,3 xylanase from position 1 to position 306 belonged to the conserved GH26 catalytic domain, and the active sites were also two glutamic acid residues (Glu-143: proton donor, Glu-231: nucleophile, labeled by a solid inverted triangle; Figure 9). Similar to the thermophilic enzyme, β-1,3 xylanase (Xyn26A), one conserved aromatic amino acid was found near the catalytic site (Tyr-142, labeled by a solid circle). Moreover, a sequence suspected of CBM containing an aromatic amino acid was observed, which can identify insoluble polysaccharide (Figure 10).

**Figure 11:**
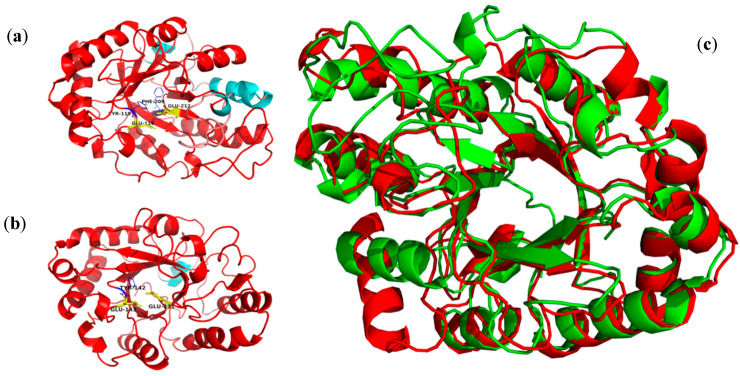
The 3D structure and alignment of the rXYL4 and Xyl88. (**a**) the 3D structure of rXYL4, (**b**) the 3D structure of Xyl88, (**c**) the 3D structure alignment, rXYL4 colored with green and Xyl88 with red.

Then, Xyl88 was modeled, analyzed, and evaluated by MolProbity. The results listed in Table S2 indicated that the modeling effect was desirable for subsequent protein structure analysis. The 3D structure of the catalytic domain with no suspected CBM sequence and that of the currently reported 1,3-xylanase (derived from rXYL4 of *Vibrio* sp. strain AX-4, PDB ID 2ddx) are shown in Figure 11. Structural alignment results showed a RMSD of 2.22, indicating that both structures were similar in the aspect of 3D structure, particularly in the active site of the enzyme, but with low sequence similarity (29.9%). Sequence similarity less than 30% always presents low reliability of modeling structure [45]. The two 3D structures were the barrel structure of the plurality of α-helices surrounding the β-folding, and the amino acid residues of the active site of the enzyme were located at the opening of the barrel. They have a conserved aromatic amino acid Tyr-14 nearby. The aromatic amino acid near the catalytic site might be related to the binding of the substrate and formation of the catalytic channel [46] (Figure S1). UCSF Chimera analyzed the hydrophilic and hydrophobic surface. The hydrophilic surface is blue and the hydrophobic surface is red in the figure. Both surfaces had strong hydrophilicity. The charge distribution on the three surfaces was analyzed by the plug-in APBS [47] installed in PyMOL. Consequently, the computational prediction model of Xyl88 had both a partial positive charge and a large amount of negative charge, whereas that of rXYL4 was electrically neutral.

Furthermore, the results of the interaction between the two intramolecular residues of the enzyme are shown in Table 3. The number of interacting residues in the Xyl88 protein structure (Nodes: 384 vs. 303) and the total number of interactions (Edges: 793 vs. 613) were significantly higher than those of rXYL4. The difference could be explained by a large number of internal hydrogen bonds (345 vs. 326) and Van der Waals force (416 vs. 249) in Xyl88. Other types of interaction were comparable. Molecular simulations of the two residues were performed at optimum temperature, and the rising temperature slightly increased the number of hydrogen bonds between rXYL4 and water (259 vs. 274). This result indicated that the room-temperature 1,3-xylanase rXYL4 structure stretched with the increase of temperature, exposing more interaction with water molecules. In contrast, the number of hydrogen bonds of thermostable Xyl88 had no significant change, indicating that its structure hardly changed, which was consistent with previous experimental results.

Finally, rapid denaturation of two enzyme molecules under high-temperature conditions (530 K and 540 K) was simulated by molecular dynamics to determine the difference of the two with regard to thermal stability.

Figure S2 shows the change of RMSD of two enzymes over time during simulation at 530 K. The RMSD of rXYL4 gradually increased, whereas Xyl88 remained unchanged. As shown in Figure S3, from the molecular simulation trajectory, the 3D structure of rXYL4 had no noticeable change in the first 6 ns, only with a flexible move in the structure. After 6 ns, the β-folding structure in the central region of the active site of the enzyme began to lose the secondary structure, and then such condition spread to the entire enzyme molecule. The fabric gradually became loose, and the peripheral α-helical structure also dissipated. By contrast, under the same conditions, the 3D structure of Xyl88 (Figure S4) did not change significantly during the whole simulation, consistent with the RMSD results. The result further proved that Xyl88 had better thermal stability than room-temperature 1,3-xylanase (rXYL4).

**Figure 12:**
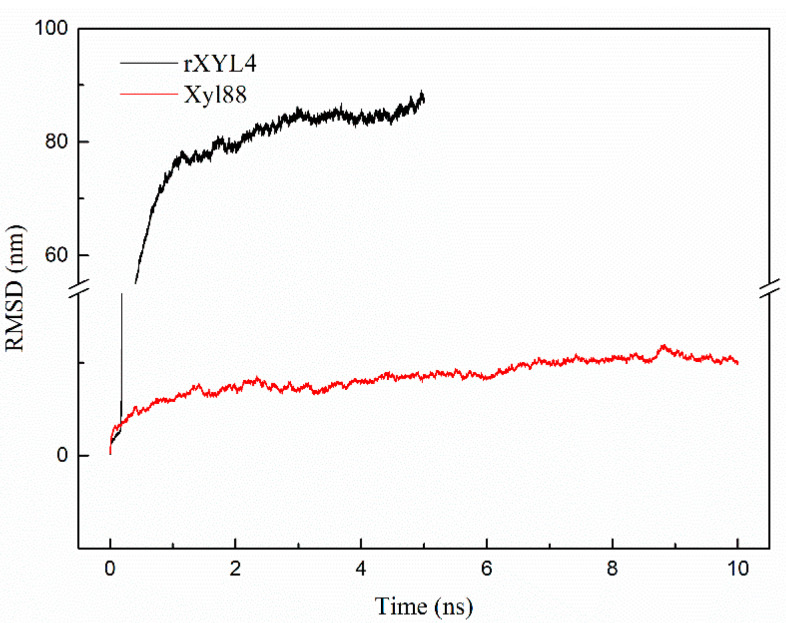
The RMSD of two β-1,3-xylanses at 540 K. Black and red lines represent the RMSD of rXYL4 and Xy188 based on the molecular dynamics simulation results for 10 ns at 540 K, respectively.

When the simulation temperature increased to 540 K, the RMSD of rXYL4 increased sharply to approximately 80 nm at 1 ns. At 5 ns, the RMSD was as high as 89.16 nm, indicating that the enzyme molecule was completely denatured (Figure 12). By contrast, the RMSD of Xyl88 only increased to approximately 6 nm in 1 ns and then gradually increased until the RMSD was close to 12 nm at 10 ns, which was smaller than the RMSD of room-temperature 1,3-xylan (rXYL4). The molecular dynamics simulation trajectory of rXYL4 (Figure S5) indicated that the structure began to lose at 0.18 ns, and the entire high-order structure of the enzyme molecule almost completely collapsed in less than 1 ns. In contrast, the β-folding structure in the central region of the active site of Xyl88 gradually disappeared from 5 ns (Figure S6), and the peripheral α-helix gradually lost its structure and became coiled irregularly. Part of the α-helix of this enzyme molecule still existed up to 10 ns, and its tertiary structure was not entirely lost, indicating that the enzyme was not totally denatured at this point. Structural changes in the catalytic center of the enzyme were important to the activities of the enzyme. The distances between catalytic residues in thermophilic enzyme Xyl88 (Glu143,Glu231) and thermophilic enzyme rXYL4 (Glu116, Glu212) were analyzed at 310 K and 540 K for 10 ns molecular dynamics simulation (Figure S7). The distances of these two enzyme were similar at 310 K (12.5 Å), but the longer distance of rXYL4 compared to that of Xyl88 after balance (40 Å vs. 32.5 Å). Although these MD results indicated that Xyl88 had significantly better thermal stability than rXYL4, low reliability of computationally predicted Xyl88 is a risk in interpreting the MD simulations.

## 4. Discussion

According to the above-mentioned experimental results, Xyl88 from the *Flammeovirga pacifica* strain was a novel moderately thermophilic salt-tolerant enzyme. Through mass spectrometry analysis, the sequence coverage was found to be 68%, probably because only trypsin was used in digestion into peptides, which resulted in insufficient enzymatic hydrolysis of the protein. In buffers without NaCl and with 1.5 mol/L NaCl, the optimum temperatures of rXyl88 were 45 °C and 55 °C, respectively, and the optimum pH was pH 7.0 under both conditions. According to the classification of thermophilic enzymes by PucciF [48], rXyl88 belonged to a moderately thermophilic enzyme and is the first reported enzyme with the temperature range among the previously reported β-1,3 xylanases listed in Table S1.

In addition, rXyl88 could tolerate up to 5 mol/L NaCl and still exhibited 80% of the highest activity at a concentration of 3 mol/L, indicating good salt tolerance. In the case of partial deactivation of rXyl88, the addition of NaCl could gradually restore its activity (Figure 7). In combination with the thermal stability results of the enzyme in Figure 6, NaCl could not only improve the thermal stability of rXyl88 but also restore rXyl88 to an active state when the enzyme was partially inactivated probably because the high electronegativity of the surface and molecular core could effectively strengthen the structure of the protein molecule and improve its thermal stability [49,50]. The salt ions might form a hydrated film-like structure on the surface of the protein that was fixed in a particular space for stability improvement [51], and Na^+^ could assist the structure in returning to the original state although the structural part was loose.

Compared with the kinetic parameters of 1,3-xylanase found in this study with other enzymes reported in the literature, rXyl88 is currently the most catalytically efficient β-1,3 xylanase, with *k_cat_/K_m_* of 314.15, nearly 20 times higher than that of the thermophilic enzyme Xyn26A reported previously [44], which had the highest catalytic efficiency (*k_cat_/K_m_* = 294.12).

The study of the effect of metal ions on enzymatic activity showed that Zn^2+^, Mn^2+^, Ni^2+^, Cu^2+^, Co^2+^, and Fe^3+^ had potent inhibitory effects on enzymatic activity, and the related enzymatic activities were all below 50%. Na^+^ (116.35%) and Mg^2^^+^ (117.63%) could improve the catalytic activity of rXyl88 to a certain extent. These two metal ions often served as the donors of similar oxidation states in the redox reaction because of their sizeable ionic radius, which led to a low charge density in the coordination sphere. Moreover, the highly flexible regulatory ligand provided good conditions for the enzyme-catalyzed bond rupture [52,53]. EDTA did not affect the activity of Xyl512 probably because rXyl88 was activated by Na^+^, whereas EDTA, as a chelating agent, only chelated polyvalent metal ions, such as Ca^2+^ and Mg^2+^, and thus had no such effect [54].

Analysis of the structure of computationally predicted Xyl88 showed that the surface had a large amount of negative charge and was highly hydrophilic. By reducing the hydrophobic surface area, the force of the high concentration salt solution was balanced to enhance the stability of the enzyme [55]. In addition, in the buffer containing NaCl, rXyl88 thermal stability was significantly improved. Studies showed that under hyper-saline conditions, the high electronegativity of the surface and molecular core could effectively enhance the structure of the protein molecule and improve the thermal stability [49,50]. Moreover, the salt ions might form a hydrated film-like structure on the surface of the protein that was fixed in a specific space to improve the stability [51], which might explain the improved thermal stability of Xyl88 in buffer containing NaCl.

In addition, Xyl88 showed a more complex interaction network between amino acid residues than room temperature 1,3-xylanase (rXYL4). The significantly increased interaction between secondary bonds improved the rigidity of enzyme molecules. This interaction made the bond more stable at high temperature, which was consistent with the properties of moderately thermophilic enzymes [56]. By calculating the number of hydrogen bonds between amino acid residues and water molecules at the optimal reaction temperatures (310 K and 328 K, respectively), the structure of rXYL4 expanded with the increase of temperature, and more amino acid residues were exposed to interact with water. Consequently, the number of hydrogen bonds between the amino acid residues and water molecules of rXYL4 significantly increased. Simultaneously, the number of hydrogen bonds formed between amino acid residues and water molecules had no significant change at these two different temperatures because Xyl88 had excellent stability, and its high-order structure remained unchanged. The results of the molecular dynamics simulations of rapid thermal denaturation of the enzyme molecules further confirmed this point.

## 5. Conclusions

The properties of the novel β-1,3 xylanase Xyl88 from *Flammeovirga pacifica* strain WPAGA1 were studied, and the obtained β-1,3 xylanase was the highest catalytic efficiency xylanase among currently reported enzymes of this family. The experimental and theoretical calculations indicated that this enzyme had moderately thermophilic and salt-tolerant properties and was the first reported β-1,3 xylanase with such unique features. The method for discovering the novel 1,3-xylanase, which was used in this paper, provided technical support for enriching the members of the β-1,3 xylanase family, which could be utilized in uncovering other enzyme molecules. The 1,3-xylanase (rXyl88) reported in this paper had high catalytic efficiency and moderately thermophilic and salt-tolerant properties. Xyl88 was suitable for the biorefinery of seaweed. Its potential for the development of seaweed energy, food and drugs needs further evaluation. Furthermore, considering the discovery of a sequence suspected of CBM in the Xyl88 sequence, the highest similarity of its sequence with the 7 CBMs of the currently reported β-1,3 xylanase was only 20.5%, and its structure and function were not yet clear. An analysis of the crystal structure of Xyl88 and related research is still required.

## Figures and Tables

**Table 1:** The influences of metal ions and chemicals on the activity of the Xyl88.

Metal Ion (10 mmol/L)	Rel Act. (%)	Metal Ion (10 mmol/L)	Rel act. (%)
Control	100.00	Cu^2+^	18.73 ± 0.73
Na^+^	116.35 ± 2.13	Ca^2+^	72.13 ± 0.62
K^+^	99.12 ± 1.29	Co^2+^	37.07 ± 0.59
Zn^2+^	35.96 ± 1.74	Ni^2+^	35.33 ± 0.84
Mn^2+^	48.54 ± 2.41	Fe^3+^	13.72 ± 0.25
Ba^2+^	106.44 ± 3.37	EDTA	73.05 ± 2.59
Mg^2+^	117.63 ± 4.16		

Rel. Act. Relative activity. Each value is the mean ± standard deviation (*n* = 3).

**Table 2:** The kinetic parameters of Xyl88 for the hydrolysis of soluble and insoluble β-1,3-xylan.

NaCl (mol·L^−1^)	Substrate	$K_{m}\;(\mathrm{mg}\cdot mL^{-1})$	$k_{cat}\;(s^{-1})$	$k_{cat}/K_{m}$
0	β-1,3-Xylan	5.75 ± 0.96	517.54 ± 41.54	90.01 ± 7.22
	Glycol β-1,3-Xylan	4.83 ± 0.64	1501.61 ± 88.41	310.89 ± 18.31
1.5	β-1,3-Xylan	5.58 ± 0.88	436.68 ± 54.81	78.26 ± 9.82
	Glycol β-1,3-Xylan	4.59 ± 0.91	1441.95 ± 130.99	314.15 ± 28.51

Data are the mean values of three independent experiments.

**Table 3:** The number of different interactions in rXYL4 and Xyl88.

3D Structure	Nodes	Edges	Protein–Protein Hydrogen Bond	Salt Bridge	Π–π Stacking	Disulphide Bond	Van der Waals	π-Cation	Protein–Water Hydrogen Bond	
									310 K	328 K
rXYL4	303	613	326	8	29	1	249	0	259	274
Xyl88	384	793	345	7	25	0	416	0	255	258

## Supplementary Materials

The following are available online at https://www.mdpi.com/2218-273X/10/9/1287/s1, Figure S1: The hydrophobic surface and electrostatic surface of the rXYL4 and Xyl88, Figure S2: The RMSD of two β-1,3-Xylanses at 530 K, Figure S3: The molecular simulation locus of rXYL4 at 530 K, Figure S4: The molecular simulation locus of Xyl88 at 530 K, Figure S5: The molecular simulation locus of rXYL4 at 540 K, Figure S6: The molecular simulation locus of Xyl88 at 540 K, Figure S7: The distance of catalytic residues of Xyl88 and rXyL4 at 310 K and 540 K, Table S1: Characteristics of the experimentally verified β-1,3-xylanases, Table S2: MolProbity evaluated results of Xyl88 structure model generated by SWISS-MODEL, Table S3: MolProbity evaluated results of Xyl88 structure model generated by I-TASSER, Table S4: MolProbity evaluated results of Xyl88 structure model generated by Robetta.

## References

[1] Hehemann J.H., Boraston A.B., Czjzek M. (2014). A sweet new wave: Structures and mechanisms of enzymes that digest polysaccharides from marine algae. Curr. Opin. Struct. Biol..

[2] Park D., Jagtap S., Nair S.K. (2014). Structure of a PL17 family alginate lyase demonstrates functional similarities among exotype depolymerases. J. Biol. Chem..

[3] Araki C. (1937). Acetylation of agar like substance of Gelidium amansii. J. Chem. Soc..

[4] Tobacman J.K. (2001). Review of harmful gastrointestinal effects of carrageenan in animal experiments. Environ. Health Perspect..

[5] Laureano-Perez L., Teymouri F., Alizadeh H., Dale B.E. (2005). Understanding factors that limit enzymatic hydrolysis of biomass. Appl. Biochem. Biotechnol..

[6] Rahman M.A., Halfar J. (2014). First evidence of chitin in calcified coralline algae: New insights into the calcification process of Clathromorphum compactum. Sci. Rep..

[7] Muzzarelli R.A. (2013). Chitin.

[8] Scheller H.V., Ulvskov P. (2010). Hemicelluloses. Annu. Rev. Plant Biol..

[9] Goddard-Borger E.D., Sakaguchi K., Reitinger S., Watanabe N., Ito M., Withers S.G.J. (2012). Mechanistic insights into the 1,3-xylanases: Useful enzymes for manipulation of algal biomass. J. Am. Chem. Soc..

[10] Macartain P., Gill C.I.R., Brooks M., Campbell R., Rowland I.R. (2007). Nutritional value of edible seaweeds. Nutr. Rev..

[11] Nguyen V.T., Ueng J.P., Tsai G.J. (2011). Proximate composition, total phenolic content, and antioxidant activity of seagrape (Caulerpa lentillifera). J. Food Sci..

[12] Coughlan M., Hazlewood G.P. (1993). Β-1, 4-smallcap D-Xylan-degrading enzyme systems: Biochemistry, molecular biology and applications. Biotechnol. Appl. Biochem..

[13] Polizeli M., Rizzatti A., Monti R., Terenzi H., Jorge J.A., Amorim D. (2005). Xylanases from fungi: Properties and industrial applications. Appl. Microbiol. Biotechnol..

[14] Maeda R., Ida T., Ihara H., Sakamoto T. (2012). Induction of apoptosis in MCF-7 cells by beta-1,3-xylooligosaccharides prepared from Caulerpa lentillifera. Biosci. Biotechnol. Biochem..

[15] Umemoto Y., Araki S.T. (2012). d-Xylose Isomerase from a Marine Bacterium, *Vibrio* sp. Strain XY-214, and d-Xylulose Production from β-1,3-Xylan. Mar. Biotechnol..

[16] Gírio F.M., Fonseca C., Carvalheiro F., Duarte L.C., Marques S., Bogel-Łukasik R. (2010). Hemicelluloses for fuel ethanol: A review. Bioresour. Technol..

[17] Granström T.B., Izumori K., Leisola M. (2007). A rare sugar xylitol. Part II: Biotechnological production and future applications of xylitol. Appl. Microbiol. Biotechnol..

[18] Tanzer J. (1995). Xylitol chewing gum and dental caries. Int. Dent. J..

[19] Iriki Y., Suzuki T., Nisizawa K., Miwa T. (1960). Xylan of siphonaceous green algae. Nature.

[20] Aoki Y., Kamei Y. (2006). Preparation of recombinant polysaccharide-degrading enzymes from the marine bacterium, *Pseudomonas* sp. ND137 for the production of protoplasts of Porphyra yezoensis. Eur. J. Phycol..

[21] Hashimoto H., Tamai Y., Okazaki F., Tamaru Y., Shimizu T., Araki T., Sato M. (2005). The first crystal structure of a family 31 carbohydrate-binding module with affinity to β-1,3-xylan. FEBS Lett..

[22] Kiyohara M., Sakaguchi K., Yamaguchi K., Araki T., Ito M. (2009). Characterization and application of carbohydrate-binding modules of β-1,3-xylanase XYL4. J. Biochem..

[23] Yamaura I., Matsumoto T., Funatsu M., Mukai E. (1990). Purification and some properties of endo-1,3-beta-D-xylanase from *Pseudomonas* sp. PT-5. Agric. Biol. Chem..

[24] Dubois M., Gilles K.A., Hamilton J.K., Rebers P.T., Smith F. (1956). Colorimetric method for determination of sugars and related substances. Anal. Chem..

[25] Kiyohara M., Hama Y., Yamaguchi K., Ito M.J. (2006). Structure of β-1,3-xylooligosaccharides generated from Caulerpa racemosa var. laete-virens β-1,3-xylan by the action of β-1,3-xylanase. J. Biochem..

[26] Kruger N.J. (2009). The Bradford method for protein quantitation. The Protein Protocols Handbook.

[27] Miller G.L. (1959). Use of dinitrosalicylic acid reagent for determination of reducing sugar. Anal. Chem..

[28] Bailey R., Bourne E.J. (1960). Colour reactions given by sugars and diphenylamine-aniline spray reagents on paper chromatograms. J. Chromatogr..

[29] Geer L.Y., Domrachev M., Lipman D.J., Bryant S.H. (2002). CDART: Protein homology by domain architecture. Genome Res..

[30] Arnold K., Bordoli L., Kopp J., Schwede T.J.B. (2006). The SWISS-MODEL workspace: A web-based environment for protein structure homology modelling. Bioinformatics.

[31] Roy A., Kucukural A., Zhang Y. (2010). I-TASSER: A unified platform for automated protein structure and function prediction. Nat. Protoc..

[32] Kim D.E., Chivian D., Baker D. (2004). Protein structure prediction and analysis using the Robetta server. Nucleic Acids Res..

[33] Chen V.B., Arendall W.B., Headd J.J., Keedy D.A., Immormino R.M., Kapral G.J., Murray L.W., Richardson J.S., Richardson D.C. (2010). MolProbity: All-atom structure validation for macromolecular crystallography. Acta Crystallogr..

[34] Delano W. (2002). Pymol Molecular Graphics System: An open-source molecular graphics tool. Ccp4 Newsl. Protein.

[35] Pettersen E.F., Goddard T.D., Huang C.C., Couch G.S., Greenblatt D.M., Meng E.C., Ferrin T.E. (2004). UCSF Chimera-a visualization system for exploratory research and analysis. J. Comput. Chem..

[36] Damiano P., Giovanni M., Tosatto S.C.E. (2016). The RING 2.0 web server for high quality residue interaction networks. Nucleic Acids Res..

[37] Sakaguchi K., Kiyohara M., Watanabe N., Yamaguchi K., Ito M., Kawamura T., Tanaka I. (2004). Preparation and preliminary X-ray analysis of the catalytic module of beta-1,3-xylanase from the marine bacterium *Vibrio* sp. AX-4. Acta Crystallogr..

[38] Phillips J.C., Braun R., Wang W., Gumbart J., Tajkhorshid E., Villa E., Chipot C., Skeel R.D., Kale L., Schulten K. (2005). Scalable molecular dynamics with NAMD. J. Comput. Chem..

[39] Humphrey W., Dalke A., Schulten K. (1996). VMD: Visual molecular dynamics. J. Mol. Graph.

[40] Lebowitz J., Percus J., Verlet L. (1967). Ensemble dependence of fluctuations with application to machine computations. Phys. Rev..

[41] Araki T., Tani S., Maeda K., Hashikawa S., Nakagawa H., Morishita T.J.B. (1999). Purification and characterization of β-1,3-xylanase from a marine bacterium, *Vibrio* sp. XY-214. Biosci. Biotechnol. Biochem..

[42] Araki T., Inoue N., Morishita T. (1998). Purification and characterization of beta-1,3-xylanase from a marine bacterium, *Alcaligenes* sp. XY-234. J. Gen. Appl. Microbiol..

[43] Kiyohara M., Sakaguchi K., Yamaguchi K., Araki T., Nakamura T., Ito M. (2005). Molecular cloning and characterization of a novel beta-1,3-xylanase possessing two putative carbohydrate-binding modules from a marine bacterium *Vibrio* sp. strain AX-4. Biochem. J..

[44] Okazaki F., Nakashima N., Ogino C., Tamaru Y., Kondo A. (2013). Biochemical characterization of a thermostable β-1,3-xylanase from the hyperthermophilic eubacterium, Thermotoga neapolitana strain DSM 4359. Appl. Microbiol. Biotechnol..

[45] Aloy P., Ceulemans H., Stark A., Russell R.B. (2003). The Relationship Between Sequence and Interaction Divergence in Proteins. J. Mol. Biol..

[46] Hamre A., Frøberg E., Eijsink V., Sørlie M. (2017). Thermodynamics of tunnel formation upon substrate binding in a processive glycoside hydrolase. Arch. Biochem. Biophys..

[47] Unni S., Huang Y., Hanson R.M., Tobias M., Krishnan S., Li W.W., Nielsen J.E., Baker N.A. (2011). Web servers and services for electrostatics calculations with APBS and PDB2PQR. J. Comput. Chem..

[48] Pucci F., Rooman M. (2017). Physical and molecular bases of protein thermal stability and cold adaptation. Curr. Opin. Biotechnol..

[49] Müllersantos M., de Souza E.M., Pedrosa F.O., Mitchell D.A., Longhi S., Carrière F., Canaan S., Krieger N. (2009). First evidence for the salt-dependent folding and activity of an esterase from the halophilic archaea Haloarcula marismortui. Biochim. Et Biophys. Acta (Bba) Mol. Cell Biol. Lipids.

[50] Elcock A.H., Mccammon J.A. (1998). Electrostatic contributions to the stability of halophilic proteins. J. Mol. Biol..

[51] Zhang T., Datta S., Eichler J., Ivanova N., Axen S.D., Kerfeld C.A., Chen F., Kyrpides N., Hugenholtz P., Cheng J.F. (2011). Identification of a haloalkaliphilic and thermostable cellulase with improved ionic liquid tolerance. Green Chem..

[52] Klee E.C.C.B. (1999). Calcium as a Cellular Regulator.

[53] Sissi C., Palumbo M. (2009). Effects of magnesium and related divalent metal ions in topoisomerase structure and function. Nucleic Acids Res..

[54] Keowmaneechai E., McClements D.J., Chemistry F. (2002). Influence of EDTA and citrate on physicochemical properties of whey protein-stabilized oil-in-water emulsions containing CaCl_2_. J. Agric. Food Chem..

[55] Siglioccolo A., Paiardini A., Piscitelli M., Pascarella S. (2011). Structural adaptation of extreme halophilic proteins through decrease of conserved hydrophobic contact surface. BMC Struct. Biol..

[56] Tompa D.R., Gromiha M.M., Saraboji K. (2016). Contribution of main chain and side chain atoms and their locations to the stability of thermophilic proteins. J. Mol. Graph. Model..

