# Supplementary material for: Identification and Characterization of a Novel Thermostable and Salt-Tolerant β-1,3 Xylanase from Flammeovirga pacifica Strain WPAGA1
Source: Biomolecules. 2020 Sep 7;10(9):1287. doi: 10.3390/biom10091287 (PMC7563424; doi:10.3390/biom10091287)
Supplement: Supplementary file 1 [file biomolecules-10-01287-s001.pdf]

Table S1 Characteristics of the experimentally verified β-1, 3-xylanases

No.	Source organism	Entry ID	pH opt	Topt/°C	Sequence Identity	R or U/ reference
1	*Flammeovirga pacifica* strain WPAGA1	A0A1S1Z2X9	7.0	45-55	100%	U/This study
2	*Thermotoga neapolitana* strain DSM 4359	B9K760	6.5	85	38.7%	U/[8]
3	*Pseudomonas vesicularis* MA103	A0A0A1C3U6	7.5	35	30.5%	U/[9]
4	*Alcaligenes sp.* strain XY-234	Q8RS40	7.5	40	30.5%	R/[10]
5	*Vibrio sp.* strain XY-214	Q9LCB9	7.0	37	30.7%	R/[46]
6	*Vibrio sp.* strain AX-4	D5MP61	7.0-7.5	37	29.9%	R/[7]

pHopt: optimal pH, Topt: optimal temperature, R: reviewed in the Uniprot Datebase, U: unreviewed in the Uniprot Datebase.

Table s2 . MolProbity evaluated results of Xyl88 structure model generated by SWISS-MODEL

All-Atom Contacts	Clashscore, all atoms:	9.88		72nd percentile* (N=1784, all resolutions)
	Clashscore is the number of serious steric overlaps (> 0.4 Å) per 1000 atoms.			
Protein Geometry	Poor rotamers	7	2.72%	Goal: <0.3%
	Favored rotamers	235	91.44%	Goal: >98%
	Ramachandran outliers	4	1.32%	Goal: <0.05%
	Ramachandran favored	283	93.71%	Goal: >98%
	MolProbity score^	2.26		61st percentile* (N=27675, 0Å - 99Å)
	Cβ deviations >0.25Å	5	1.82%	Goal: 0
	Bad bonds:	0 / 2538	0.00%	Goal: 0%
	Bad angles:	27 / 3452	0.78%	Goal: <0.1%
Peptide Omegas	Cis Prolines:	0 / 10	0.00%	Expected: ≤1 per chain, or ≤5%
	Cis nonProlines:	1 / 293	0.34%	Goal: <0.05%

Table s3. MolProbity evaluated results of Xyl88 structure model generated by I-TASSER

All-Atom Contacts	Clashscore, all atoms:	14.55		51st percentile* (N=1784, all resolutions)
	Clashscore is the number of serious steric overlaps (> 0.4 Å) per 1000 atoms.			
Protein Geometry	Poor rotamers	71	19.45%	Goal: <0.3%
	Favored rotamers	221	60.55%	Goal: >98%

	Ramachandran outliers	67	15.84%	Goal: <0.05%
	Ramachandran favored	279	65.96%	Goal: >98%
	MolProbity score^	3.52		8th percentile[*] (N=27675, 0Å - 99Å)
	Cβ deviations >0.25Å	62	16.02%	Goal: 0
	Bad bonds:	4 / 3484	0.11%	Goal: 0%
	Bad angles:	151 / 4735	3.19%	Goal: <0.1%
Peptide Omegas	Cis Prolines:	1 / 13	7.69%	Expected: ≤1 per chain, or ≤5%
	Cis nonProlines:	2 / 411	0.49%	Goal: <0.05%
	Twisted Peptides:	69 / 424	16.27%	Goal: 0

Table s4. MolProbity evaluated results of Xyl88 structure model generated by Robetta

All-Atom Contacts	Clashscore, all atoms:	0.45		99th percentile[*] (N=1784, all resolutions)
	Clashscore is the number of serious steric overlaps (> 0.4 Å) per 1000 atoms.			
Protein Geometry	Poor rotamers	0	0.00%	Goal: <0.3%
	Favored rotamers	362	99.18%	Goal: >98%
	Ramachandran outliers	4	0.95%	Goal: <0.05%
	Ramachandran favored	389	91.96%	Goal: >98%
	MolProbity score^	1.15		99th percentile[*] (N=27675, 0Å - 99Å)
	Cβ deviations >0.25Å	0	0.00%	Goal: 0
	Bad bonds:	4 / 3484	0.11%	Goal: 0%

	Bad angles:	11 / 4735	0.23%	Goal: <0.1%
Peptide Omegas	Cis Prolines:	0 / 13	0.00%	Expected: ≤1 per chain, or ≤5%

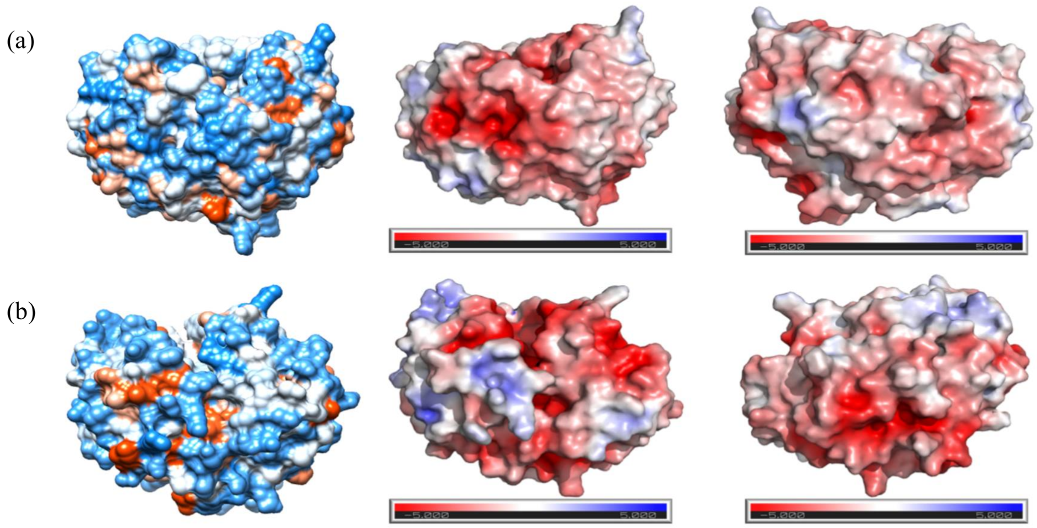

Figure. S1 The hydrophobic surface and electrostatic surface of the rXYL4 and Xyl88.

a: rXYL4, b: Xyl88

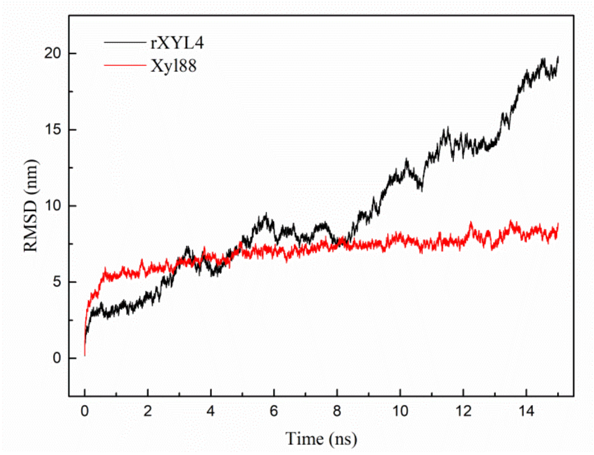

Figure S2 The RMSD of two β-1,3-Xylanses at 530 K.

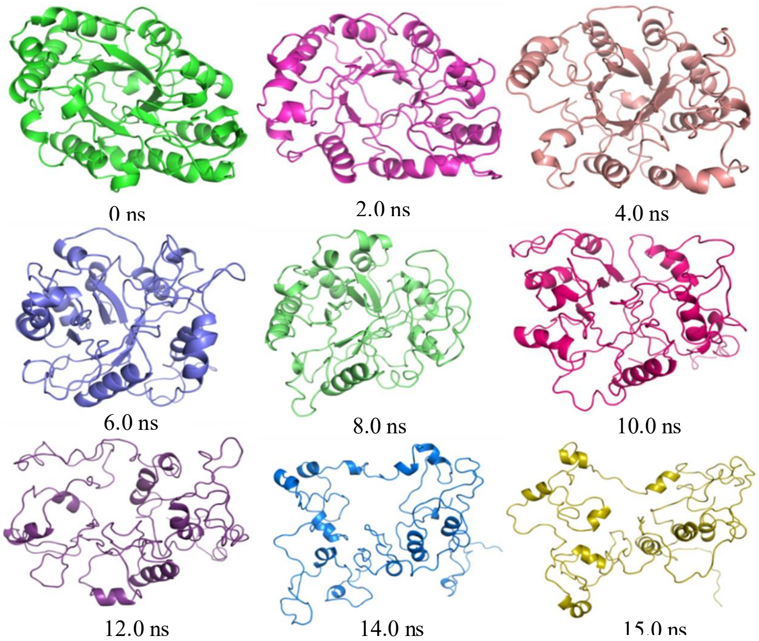

Figure S3 The molecular simulation locus of rXYL4 at 530 K

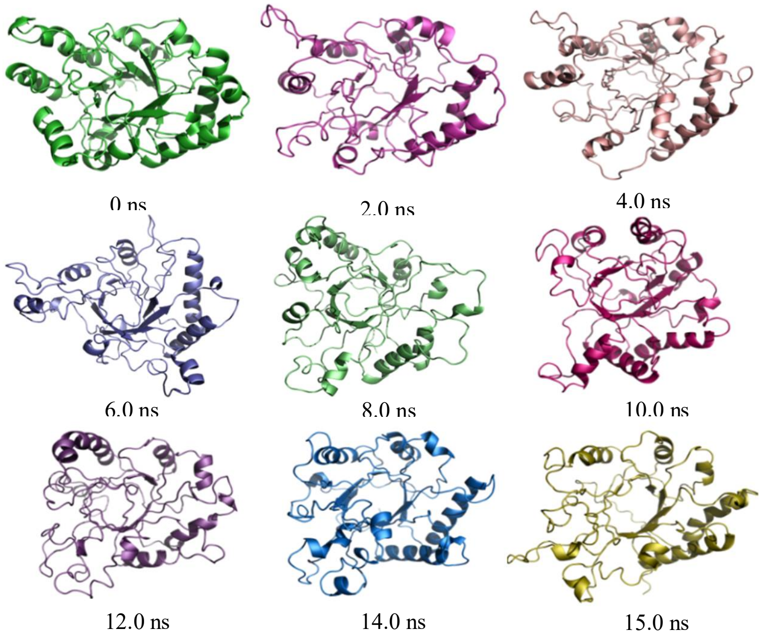

Figure S4 The molecular simulation locus of Xyl88 at 530 K

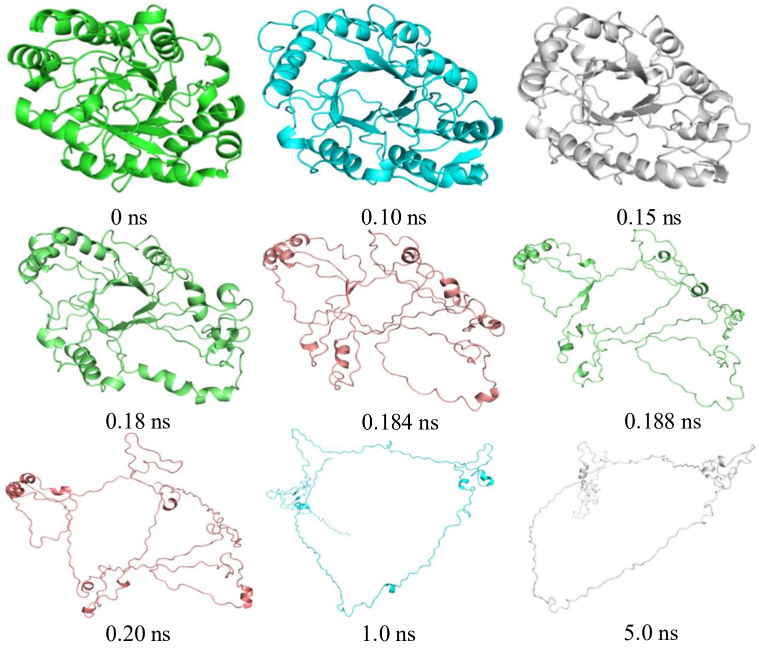

Figure S5 The molecular simulation locus of rXYL4 at 540 K

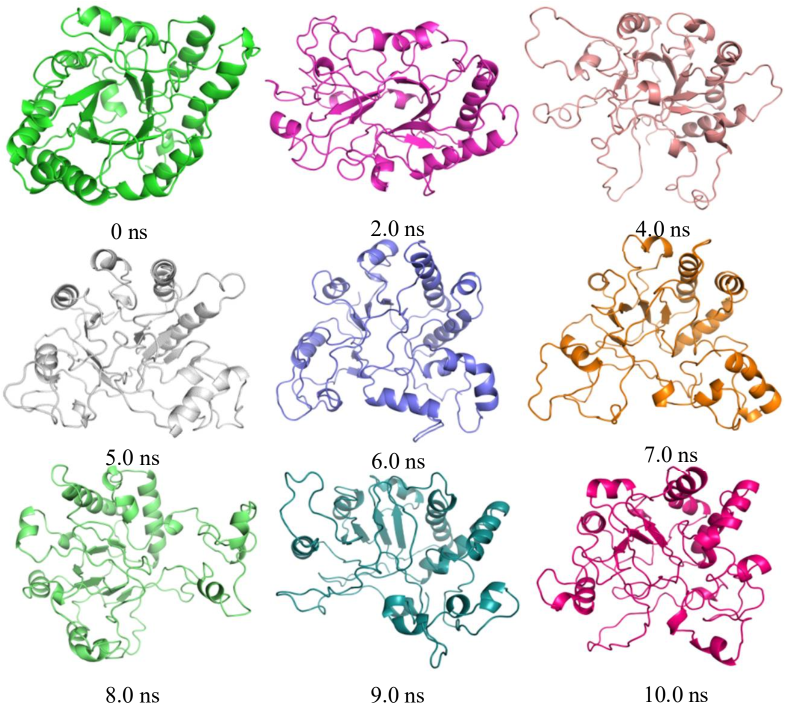

Figure S6 The molecular simulation locus of Xyl88 at 540 K

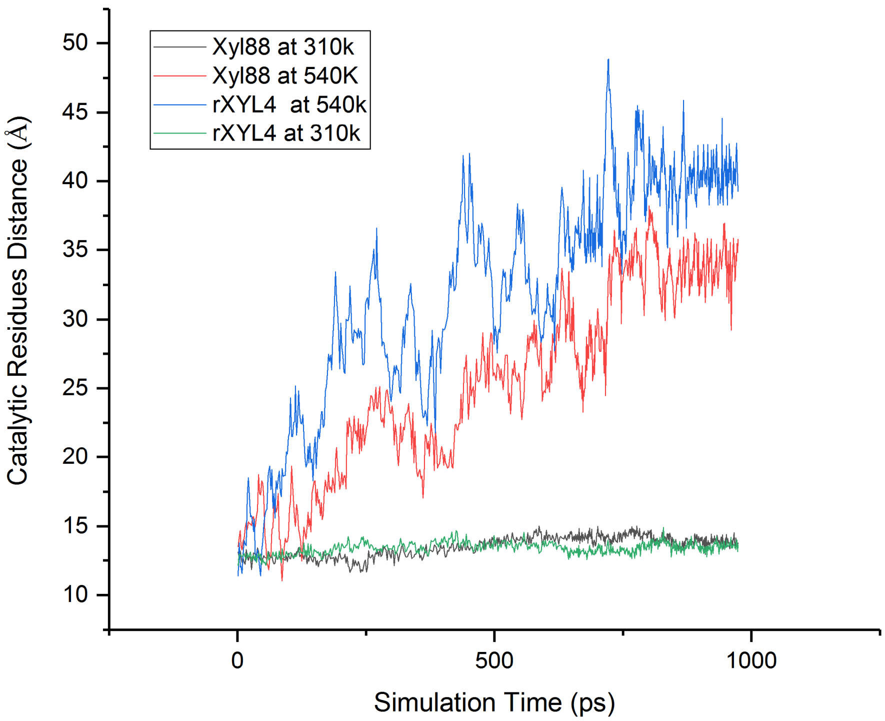

Figure S7 The distance of catalytic residues of Xyl88 and rXyL4 at 310K and 540 K
